# Supplementary material for: Learning curve and surgical outcome of robotic assisted colorectal surgery with ERAS program
Source: Sci Rep. 2022 Nov 29;12:20566. doi: 10.1038/s41598-022-24665-w (PMC9709162; doi:10.1038/s41598-022-24665-w)
Supplement: Supplementary file 2 — Supplementary Information 2. [file 41598_2022_24665_MOESM2_ESM.zip › RAL Raw data-Ñ[▒K/076/HS_ñw╝╨░O▒Kñσ.pdf]

正式摘要(電子簽章：000000215575839)

W52 / W52 ☐ 英文標題(注意:無英文姓名-請補病人基本檔!) ☐ 【讀取】憑證

臺中榮民總醫院

出院病歷摘要

轉入號 : 轉出號 :

[redacted]

(8)流水編號	
(9)入院日期	110/04/14 (2021) 直腸外科 W52 - 145
(10)轉科日期	
(11)出院日期	110/04/25 (2021) 住院天數計 11 日

診斷

- 入院 :S colon tumor 15 cm FAV
- 出院 :S colon tumor, 15 cm FAV
  s/p robotic AR 2021/4/21.
  pTx,Nx.

主訴

- bloody stool for 3m.

病史

- This is a 84-year-old female with history of:
  1. DM
  2. HTN
  3. CAD s/p stent 2020/7
  According to the patient, she suffered from bloody stool. COS proved S colon ca 15 cm FAV. She visited CRS OPD and was admitted for LAR. She is totally ADL independant. She denies any allergy or family history.

體檢發現

- CEA

  2021/4 CT: S colon ca.
  - General appearance :
    - easy looking , conscious clear , without cardiopulmonary distress
    - Vital signs自動帶入date 體溫(BT) 脈搏(P) 呼吸(R) 血壓(BP) 身高(BH) 體重(BW) BMI
      20210414 36.8℃ 94次/分 18次/分 163/87mmHg 157.7cm 74.6kg 30.0
    - Vital signs: B.P. 163/87 mmHg , P.R. 94 /min , R.R. 18 /min , B.T. 36.8 ℃
  - HEENT :
    - Eyes: no anemic conjunctiva, no icteric sclera, normal EOM
    - ENT: no otorrhea, no rhinorrhea, no erythematous mucosa, no injected tonsil, no deformity
  - Neck: supple, freely movable, trachea midline,no deformity, no engorged jugular vein , no palpable thyroid or lymph node enlargement
  - Chest:
    - Inspection: Grossly normal, symmetric and fully expansion, no accessory muscle used, no operation scar, no spider angiomata, no gynecomastia
    - Palpation: no palpable mass or nodule, no subcutaneous emphysema, no tenderness
    - Ausculation: clear breathing sound
  - Heart:
    - Percussion: normal heart size
    - Ausculation: regular heart beat, normal S1, S2, no gallop, no murmur
  - Abdomen:
    - Inspection: in

-
    - flat contour, symetrically, no scar, no stria, no dilated veins, no rashes
  - Ausculation: normoactive bowel sound, no abdominal bruits
  - palpation: soft, no muscle guarding or rigidity, no local tenderness, no rebounding pain, no palpable mass, no palpable pulsation
  - Percussion: no shifting dulllness, no hepatomegaly, no splenomagaly
-

Peripheral pulsation :	CA	BA	RA	FA	PA	PTA	DPA
Right side :	++	++	++	++	++	++	++/+++
Left side :	++	++	++	++	++	++	++/+++

- Genito-anus: no tenderness or palpable mass lesion or rectal shelf, normal external genitalia, normal anal tone, brown stool

CRS CONDITION

-
  - Lung

手術日期及方法

- 手術日期及方法20210421 Robot Assisted Colectomy(L)

住院治療經過

- The patient was admitted for robotic AR. Due her partial obstruction, PPN was applied. PET for tumor staging without metastasis noted. We initiated nutrition support and ERAS protocol. She underwent surgery on 2021/4/21. After surgery, she went back to ward for post-operative care. She recovered smoothly under ERAS protocol. After oral intake smoothly and educated home care. MBD and OPD f/u.

合併症

一般檢查

特殊檢查

病理報告

其它

出院時情況

- 3.改門診治療

出院指示

藥名	劑量	劑量單位	用法	頻次	數量	數量單位	現況	天數	單位
Voren-K tab 25mg	1	TAB	PO	TID	15	TAB	TH	5	天
(□)Takepron OD tab 30mg	1	TAB	PO	QDAC	5	TAB	TH	5	天
Ulstop tab 20mg	1	TAB	PO	TIDPRN	15	TAB	TH	5	天
Ultracet tab	1	TAB	PO	Q6H	20	TAB	TH	5	天
500mg Glucophage	1	TAB	PO	BID	10	TAB	TH	5	天
5/160mg Exforge tab	1	TAB	PO	QD	5	TAB	TH	5	天
Acetylcysteine tab	1	TAB	PO	QN	5	TAB	TH	5	天
Mag. Oxide tab	2	TAB	PO	TID	30	TAB	TH	5	天

出院計劃

主治醫師 2222H 林俊余 / 住院醫師 2222H 主治 林俊余

正式摘要(電子簽章：**000000215575839)**

W52 / W52

doctorRank:B
